# Effect of guided imagery relaxation on anxiety in cervical cancer: randomized clinical trial

**DOI:** 10.1590/0034-7167-2021-0874

**Published:** 2023-10-06

**Authors:** Edenice de Oliveira Santana, Leonel dos Santos Silva, Luana Aparecida Alves da Silva, João Lucas de Aquino Lemos, Larissa Marcondes, Paulo Ricardo Bittencourt Guimarães, Luciana Puchalski Kalinke

**Affiliations:** I Universidade Federal do Paraná. Curitiba, Paraná, Brazil

**Keywords:** Uterine Cervical Neoplasms, Imagery, Psychotherapy, Relaxation Therapy, Anxiety, Radiotherapy, Neoplasias del Cuello Uterino, Imágenes en Psicoterapia, Terapia por Relajación, Ansiedad, Radioterapia, Câncer de Colo do Útero, Imagens, Psicoterapia, Terapia de Relaxamento, Ansiedade, Radioterapia

## Abstract

**Objectives:**

to evaluate the effect of guided imagery relaxation through virtual reality on anxiety in women with cervical cancer undergoing radiochemotherapy.

**Methods:**

randomized, non-blinded, single-center clinical trial conducted at a cancer reference hospital. 52 women participated, with randomized allocation of 24 in the control group and 28 in the experimental group (12 sessions of guided imagery relaxation through virtual reality, applied three times a week). The outcome was evaluated using the State-Trait Anxiety Inventory and statistical analysis was performed using the Generalized Linear Mixed Model.

**Results:**

n the experimental group, women presented significant anxiety traits (p=0.010) before the intervention. Between the 4th and 12th week of follow-up, there was a reduction in anxiety levels, without statistical significance.

**Conclusions:**

guided imagery relaxation through virtual reality provided evidence of anxiety reduction in women with cervical cancer undergoing radiochemotherapy and may contribute to clinical practice. Brazilian Clinical Trial Registry: RBR-7ssvytb.

## RESUMO

**Objetivos:**

avaliar o efeito do relaxamento com imagem guiada por realidade virtual na ansiedade em mulheres com câncer cervical submetidas à radioquimioterapia.

**Métodos:**

ensaio clínico randomizado, não mascarado, unicêntrico, realizado em um hospital de referência em câncer. Participaram 52 mulheres com alocação randomizada, 24 no grupo controle e 28 no experimental (12 sessões de relaxamento por imagem guiada por realidade virtual, aplicadas 3 vezes por semana). O desfecho foi avaliado pelo Inventário de Ansiedade Traço-Estado e a análise estatística foi realizada com o *Generalized Linear Mixed Model*.

**Resultados:**

no grupo experimental, as mulheres apresentavam traços de ansiedade significativos (p=0,010) antes da intervenção. Entre a 4ª e 12ª semana de seguimento, houve redução no estado de ansiedade, sem significância estatística.

**Conclusões:**

a técnica de relaxamento por imagem guiada por realidade virtual forneceu evidências de redução da ansiedade em mulheres com câncer cervical em tratamento com radioquimioterapia e pode contribuir na prática clínica. Registro Brasileiro de Ensaio Clínico: RBR-7ssvytb.

## RESUMEN

**Objetivos:**

evaluar el efecto de la relajación con imagen guiada por realidad virtual en la ansiedad en mujeres con cáncer cervical sometidas a radioterapia.

**Métodos:**

ensayo clínico aleatorizado, no enmascarado, unicéntrico, realizado en un hospital de referencia en cáncer. Participaron 52 mujeres con asignación aleatoria, 24 en el grupo control y 28 en el experimental (12 sesiones de relajación por imagen guiada por realidad virtual, aplicadas 3 veces por semana). El resultado fue evaluado por el Inventario de Ansiedad Rasgo-Estado y el análisis estadístico fue realizado con el *Generalized Linear Mixed Model*.

**Resultados:**

en el grupo experimental, las mujeres presentaban rasgos de ansiedad significativos (p=0,010) antes de la intervención. Entre la 4ª y 12ª semana de seguimiento, hubo reducción en el estado de ansiedad, sin significancia estadística.

**Conclusiones:**

la técnica de relajación por imagen guiada por realidad virtual proporcionó evidencias de reducción de la ansiedad en mujeres con cáncer cervical en tratamiento con radioterapia y puede contribuir en la práctica clínica. Registro Brasileño de Ensayo Clínico: RBR-7ssvytb.

## INTRODUCTION

Cervical cancer (CC) is a socially and scientifically distinguishable malignant tumor that poses a global health challenge as the fourth leading cause of cancer in women. Controlling CC is tragically associated with weaknesses at different levels of healthcare. Actions such as vaccination against human papillomavirus, screening, treatment of precursor lesions, and early diagnosis are global strategies to control the disease by 2030^(1)^.

Globally, in 2020, 604,127 new cases and 341,831 deaths were estimated, with more than 90% occurring in developing countries^(1-3)^. In the same period, the incidence of 16,710 new cases and 10,191 deaths ranked third as a cause of death among Brazilian women. CC treatment and prognosis are based on staging, with surgical interventions, concurrent radiochemotherapy, and immunotherapy being indicated^(3-4)^.

These treatments affect sexual function and may reduce shortand long-term health-related quality of life (HRQoL). Moreover, they can result in hormonal, immunological, bladder, urinary, gastrointestinal, lymphedema, pain, anxiety (with variable prevalence ranging from 20 to 65%), depression, low self-esteem, fear, body image disturbances, social isolation, and marital tension, which are among the most frequent issues experienced by these women^(3,5-6)^.

Anxiety and other psychological distress are prevalent in modern societies and women affected by CC are at higher risk of mortality^(7-8)^. It is an individual feeling about an uncertain future, an unpleasant emotion caused by intrusive thoughts. It results in positive or maladaptive behaviors, with physical and mental dysfunctions, which can be alleviated with feasible integrative practices without side effects, either in active treatment, palliative care, or survivorship^(9-12)^.

Among the available integrative practices, guided imagery relaxation therapy is a low-cost, safe, and easily applicable intervention. For this study, relaxation and guided imagery therapies were used, applied with virtual reality. Physiologically, relaxation acts on the excitatory reduction of the sympathetic nervous system and stress levels, consequently presenting a positive influence on psychoneuroimmunology^(13)^. Guided imagery is a mind-body integrative practice that uses mental visualization to promote neurophysiological responses, with the aim of balancing and improving physical and mental symptoms. When used concomitantly with drug treatment, it can improve QoL, immune function, muscle relaxation, pain, fatigue, dyspnea, nausea, vomiting, anxiety, depression, and mood^(11-18)^.

Relaxation with guided imagery (RGI) involves systematically tensing and relaxing different muscle groups through controlled breathing, while visualizing nature images that trigger anxiolytic effects. However, accessing such images may not always be feasible in different social contexts^(14,18)^. Virtual reality (VR) can serve as a resource associated with RGI to facilitate the immersive conduction of the participant in different scenarios, as a way of overcoming physical access to nature^(19)^. It is a simulation technology capable of providing 3D visualization that promotes a highly realistic and interactive experience in different scenarios; it can aid in relaxation and changes in physiological markers. It is a viable intervention that can be applied in the clinical environment of oncology patients as an effective non-pharmacological adjunct^(19)^.

Given the above, the study is justified for oncology nursing practice in the proposition of effective and feasible interventions in the care of patients with cervical cancer. Furthermore, the incipiency of research for this vulnerable population, who suffer from anxious symptoms resulting from the disease or treatment, is clarified in research that underlies the proposition of RGI in this study^(11,20-21)^.

## OBJECTIVES

To evaluate the effect of relaxation with guided imagery through virtual reality on anxiety in women with cervical cancer undergoing radiochemotherapy.

## METHODS

### Ethical considerations

The research was approved by the research ethics committee and was in accordance with the Brazilian Resolution 466/2012 for research involving human subjects. The clinical trial was registered (RBR-7ssvytb) in the Brazilian Registry of Clinical Trials.

### Study design, period, and location

A randomized, non-masked, single-center clinical trial was conducted at the radiation therapy service of an exclusive hospital for cancer treatment, a reference in the South of Brazil, from October 2019 to January 2021. The study design was guided by the Consolidated Standards of Reporting Trials (CONSORT) recommendations.

### Population or sample, inclusion and exclusion criteria

Fifty-two women with cervical cancer who were recently admitted to the radiation therapy service were included in the study. Sample size calculation (71 participants) based on simple dimensioning was based on the mean (86 cases) of women who underwent radiochemotherapy from 2016 to 2018, with a margin of error of 5% and confidence level of 95%. Inclusion criteria were age over 18 years and indication of treatment for cervical cancer with radiochemotherapy.

Exclusion criteria included hospitalized women, those undergoing pelvic re-irradiation, those using antidepressants and psychotropic drugs, those with neurological deficits (motor or cognitive deficits recorded in the medical record), and those using integrative practices (such as yoga, meditation, among others). Discontinuation criteria were: initiation of antidepressants and anxiolytics during the study period, interruption of radiochemotherapy or failure to complete the intervention protocol sessions. However, there was no loss of follow-up and no reports of adverse events, side effects, or protocol deviations.

### Study Protocol

The randomization process was performed using sealed envelopes containing the acronyms GE (experimental group) and GC (control group), numbered from 01 to 71 (36 for GE and 35 for GC) and delivered randomly. After eligibility evaluation and consent signing, participants chose one of the envelopes and opened it to identify their allocation group. Due to the COVID-19 pandemic, recruitment was interrupted, and data collection occurred from October 2019 to January 2021. Therefore, 28 women were randomized in the GE and 24 in the GC.

Participants in both groups underwent standard treatment: weekly chemotherapy applications, five teletherapy sessions per month, and four brachytherapy insertions per month. In addition, they were followed up during the first medical and nursing appointments and then in weekly consultations until discharge. The GE received the intervention (RGI by VR) for 12 sessions^(14,18-19)^, while the GC received standard treatment. Both groups were followed up for 28 days.

The intervention performed was the RGI by VR technique, applied three times a week. Participants were directed to a reserved, quiet room and comfortably positioned in a chair or stretcher (preferably individual). They were then invited to choose one of the available videos (sunset, moonlight, sitting on a beach, floating in water, or space). Participants were then instructed on how to handle the VR glasses control (BOBO VR Z4^®^ brand), with high-quality headphones and 3D movement. The 360º videos were edited in Adobe Premiere Pro CC 2018^®^ and lasted nine minutes and 52 seconds.

The technique begins with the video on a dark screen for initial relaxation (five minutes and 25 seconds). The audio invites the participant to perform breathing exercises and muscle movements that induce a state of relaxation. Then, the guided image with sensory guidance (four minutes and 14 seconds) is introduced, such as immersing in nature, interacting with smells, temperature, textures, sounds, and sensations with one’s body, and alternating slow and deep breathing. In the last 13 seconds, the participant is directed to pay attention to the sensations, focus on the rhythm of their breathing, and calmly and peacefully return to the environment.

All participants and sessions were directly supervised and conducted by the same researcher to ensure protocol uniformity. The equipment was protected with adherent polyvinyl chloride film to avoid direct contact with participants’ skin without compromising audiovisual quality. After use, disinfection was performed.

For analysis of population characteristics, after randomization, an instrument was applied to assess secondary variables (sociodemographic and clinical), such as age, occupation, marital status, staging, comorbidities, among others.

### Outcome

For the primary outcome variable assessment, the State-Trait Anxiety Inventory (STAI) and the State Anxiety Inventory (STAI-S), developed by Spielberger, Gorsuch, and Lushene (1970)^(22)^, and translated and adapted for Brazil with satisfactory internal consistency (Cronbach’s alpha between 0.87 and 0.93)^(23)^, were applied. The STAI-T and STAI-S are two distinct self-evaluation subscales, each with 20 statements for each concept (trait, general feeling, and state, determined moment or situation). They are scored from 1 to 4 (very much to absent), and scores range from 20 to 80 (minimum to maximum). The population mean is 40, with >42 indicating anxiety and <38 indicating depression^(21-23)^. Both inventories were applied at the baseline assessment.

During the follow-up period, the STAI-S instrument was applied to both groups three times a week (a total of 12 assessments) over a 28-day period. In the experimental group, the application occurred after the intervention, and in the control group, after conventional treatment. The intervention and data collection were performed in a reserved room before radiotherapy sessions. The time for filling out the instruments was approximately 5 to 8 minutes before the intervention began.

### Data Analysis

All obtained data were double-digitized, independently and subsequently validated. The responses were coded into Microsoft Excel Office 365^®^ spreadsheets. The Statistical software Statistica^®^ version 7 with double-entry was used for data analysis. The Generalized Linear Mixed Model was adopted for the primary variables of the groups and assessments, applied when the measures do not need to be equally spaced and balanced^(24)^. The confirmation of the significance analysis was performed by the Sidak’s test (p>=0.05). The model adjustment was defined by the Akaike’s Information Criterion (AIC), and the autoregressive covariance matrix of order 1 (AR1) was used. The secondary variables were submitted to descriptive analyses, and the homogeneity of the samples was analyzed by Mann Whitney tests for age and chi-square for the other variables.

## RESULTS

**Figure 1:**
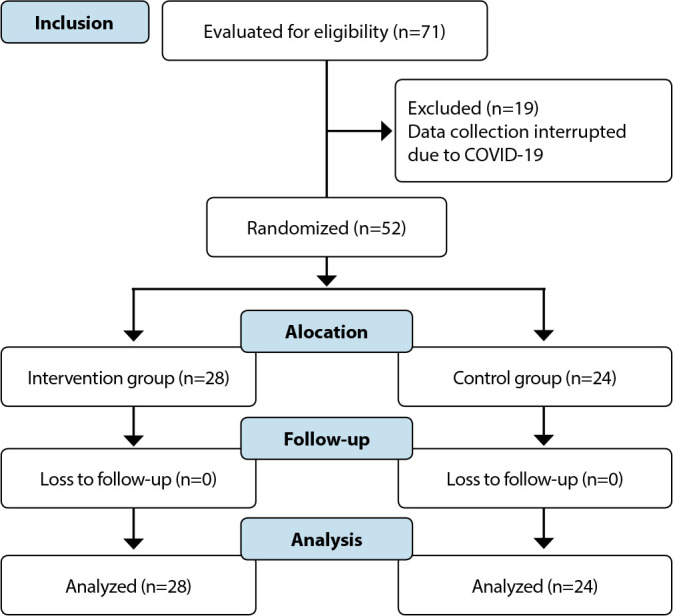
Recruitment and allocation flowchart of study participants, Curitiba, Paraná, Brazil, 2021

Recruitment occurred from October 2019 to January 2021, with 28 women randomized to the Experimental Group and 24 to the Control Group (Figure 1).

Among the sociodemographic and clinical characteristics (Table 1) for both the CG and EG, it is noteworthy that the mean age was 41.1 and 48.4 years, respectively, with differences in marital status and education level between the groups. Both groups were similar in terms of advanced clinical staging, presence of comorbidities, and predominantly sedentary lifestyle. Sample homogeneity regarding variable distribution was observed between the groups after randomization (Table 1).

**Table 1:** Sociodemographic and clinical characteristics of women with cervical cancer undergoing radiochemotherapy, randomized to control and experimental groups, Curitiba, Paraná, Brazil, 2021

Variable	Control group (n=24)		Experimental Group (n=28)		*p* ^*^
	n	(%)	n	(%)	
Age in years (mean)	41.2		48.4		0.16
Marital status					
Single	10	41.67	09	32.14	
Married/stable union	07	29.17	12	42.85	0.59
Separated/divorced/widowed	07	29.16	07	25.01	
Education					
Up to elementary school	07	29.17	14	50	
High school	14	58.33	11	39.29	0.30
Higher education	03	12.50	03	10.71	
Employment status					
Active/homemaker	15	62.50	18	64.29	0.88
Retired/unemployed	9	37.50	10	35.71	
Income					
Up to 3 minimum wages	23	95.83	24	85.71	0.45
3 to 10 minimum wages	01	4.17	04	14.29	
Staging					
Early (IB1, IB2, IIB and IIA)	11	45.83	11	39.29	0.84
Advanced (IIIB, IIIA, IIIC, IVB and IVA)	13	54.17	17	60.71	
Comorbidities					
Present	13	50.17	15	53.57	0.81
Sedentary lifestyle					
Present	23	95.8	28	100	0.94

n - number of participants,

* p=<0.05.

In the evaluation of the Trait Anxiety variable (IDATE-T), it was demonstrated that the patients presented traits of anxiety prior to the intervention. In the EG, the mean score was 42.11, higher than the CG with a mean of 36.61, and this previous difference was statistically significant (p=0.01) (Table 2).

**Table 2:** Comparison of anxiety traits (IDATE-T), Curitiba, Paraná, Brazil, 2021

Group	n	Average	SD	Minimum	Maximum	*p*
Experimental	28	42.11	6.49	28	53	0.01^*^
Control	24	36.61	8.42	17	51	
Total		39.63	7.85	17	53	

n - number of participants; SD - Standard Deviation; p: <0.05 statistical significance.

**Figure 2:**
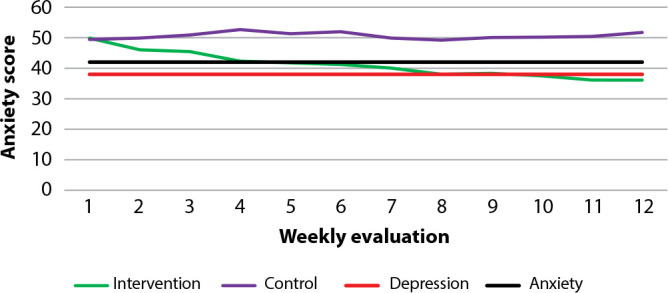
Comparison of mean state anxiety scores (IDATE-E), Curitiba, Paraná, Brazil, 2021

When evaluating anxiety state (IDATE-E), we identified a baseline mean of 49.86 in the EG and 49.20 in the CG, with a decline in scores in the EG and an increase in the CG from the second assessment onwards (Figure 2).

From the fourth to the twelfth assessment, there was an increase in anxiety in the CG and a significant decrease in the EG, which occurred up to the limit of depression (EG), but was not considered significant (Table 3/Figure 2).

**Table 3:** Statistical model for the dependent variable state anxiety (IDATE-E), Curitiba, Paraná, Brazil, 2021

Source	Type III Fixed Effects Tests			
	n	DF	F	*p*
Intercept	1	69.077	4436.126	0.00
Group	1	69.077	48.353	0.00^*^
Time	2	348.429	1.760	0.06
Group ^*^ time	2	348.429	2.455	0.00

N - numerator; DF: degree of freedom - F: Snedecor's F-statistic; p: <0.05, statistical significance; Akaike's Information Criterion (AIC) = 3453.95.

## DISCUSSION

Women with cervical cancer suffer from the aggressiveness of radiochemotherapy, changes in daily life activities, and live with anxiety throughout the therapeutic course. Therefore, the healthcare team should be attentive to these modifications and offer interventions that minimize anxiety and provide overall well-being.

Our findings corroborate with the profile of women from other continents affected by CC: age between 40 and 49 years old and in the workforce. The socioeconomic impact generates negative feelings of stress, anxiety, and delay in diagnosis^(3,25)^. The presence of a partner is associated with a lower incidence of anxiety, early detection, and better prognosis. In unmarried women, there is an increased risk of delayed diagnosis, lower survival, and economic and educational insufficiency related to delayed treatment^(8,12,26-27)^.

Radiochemotherapy in advanced disease (IIB-IVA) presents a worse prognosis, with improved survival in 5 years, prevalence of genitourinary sequelae, higher toxicity in elderly individuals, lower treatment adherence, limitations for surgical treatment indication, and decline in QoL. The presence of comorbidities and sedentary lifestyle results in a higher incidence of psychiatric symptoms and risk of mortality^(11-12,25,28-29)^.

We emphasize the importance of identifying risk factors for anxiety in these women, understanding that they are a vulnerable group for developing psychological distress, and implementing screening measures for anxious and depressive symptoms in the care process. Therefore, multidisciplinary actions associated with qualified care are fundamental in empowering women to access services, promote therapeutic adherence, and improve overall health conditions^(30)^.

Our initial findings showed traces of anxiety (i.e., a stable variable indicating a greater disposition to feel anxious) prior to the inclusion of participants in the research, possibly related to the sociodemographic, clinical, and therapeutic factors of CC. It is worth noting that depression and anxiety are associated with a lower probability of screening^(31)^ and delays in diagnosis, which justifies the higher number of participants with advanced disease. They compromise treatment adherence and affect QoL^(21)^. Sociocultural issues may impact the under-recognition of anxiety, reluctance to allopathic and psychotherapeutic treatment, and aggravate the clinical condition^(21)^.

During RGI sessions, the reduction of anxiety state (moment or particular situation related to the procedures, for example) can be explained by the neurophysiological effect of the intervention. Some of the sessions occurred during the period of greatest discomfort, fear, and anxiety due to brachytherapy (intracavitary application of ionizing radiation). Our data are corroborated by a systematic review that analyzed the outcome of RGI in women with breast cancer, showing a reduction in anxiety and depression, improved mood, body discomfort, and long-term improvement of HRQoL. However, regarding the effects of chemotherapy, such as nausea and vomiting, pain, and fatigue, better evidence is needed^(32)^.

The RGI intervention is psychologically favorable and increases comfort in cancer treatment. It is effective in mental state, reducing average scores of depressions, anxiety, and biomarker effects (heart rate, blood pressure, cortisol, amylase, and immunity)^(15-16,18,20)^. In hospitalized patients for pain control, it did not show an effect on pain scores but reduced anxious symptoms and opioid consumption with statistical significance^(21)^.

Different practices can be used concurrently with guided imagery. Music therapy was applied in heterogeneous populations of cancer patients, showing a significant reduction in trait-state anxiety levels^(33)^. The combination of music, guided spiritual images, and muscle relaxation had an effect on psychological distress (stress, anxiety, and depressive symptoms), coping, and resilience scores in cancer patients^(34)^.

Confirming our hypothesis, women with anxiety, either state or trait, presented a reduction during RGI sessions, concomitant with radiochemotherapy. We believe that the immersive potential of the intervention, made possible by VR^(19,28)^, increases cognitive distraction. Since state anxiety is transient and situational, both measurement and intervention were performed at the moment of greatest rumination of feelings about treatment. It makes sense that these women would respond to the intervention, which was not observed in the control group.

We noticed curiosity, acceptability, and adherence (not evaluated in this research) to the use of VR for RGI application. We reflect that, for future issues in different care environments, they could even be self-applied by patients when well-guided. It is an immersion mediated by electronic devices with the aid of smartphones, which are already widely used in society, and VR has been a growing object of study for different variables, clinical contexts, and heterogeneous populations^(19,28)^.

Given the results of this and other related research, the use of the technique is recommended as a complementary means to minimize anxiety, as this suffering can present itself before, during, and extend after treatment. To do so, it would be necessary to expand the network of care with a psychosocial focus and train professionals in the implementation of cognitive-behavioral skills. It is a simple and low-cost intervention^(20-21,32)^.

### Study limitations

This research presents limitations that should be considered. Among them, we highlight the interruption of data collection due to the sanitary conditions imposed by COVID-19. Given the characteristics of the intervention, we did not perform participant or researcher masking, and we believe that future research could be developed with a pseudonymized or masked intervention. It is possible that participants who receive psychotherapeutic follow-up (although it was not provided in the service where the research was carried out) may have represented a risk of bias in the results, which was not controlled for. Psychometric assessments were not correlated with biomarkers and/or qualitative analyses to corroborate the effects and feelings resulting from the intervention.

### Contributions to the Nursing, Health, or Public Policy Areas

Our study contributes significantly to the field of nursing by demonstrating complementary techniques for clinical practice, as well as promoting the autonomy of professionals in implementing safe and low-cost interventions. In addition to the psychometric evidence of our findings, we highlight the frequent satisfactory reports from participants (not included in the scope of this research). We reflect that it is a viable intervention that could be made protocol available in this and other institutional realities.

For the health field, we highlight the possibilities of promoting quality of life by minimizing the deleterious effects of anxiety on the health of women with cervical cancer undergoing radiochemotherapy treatment. In the future, pragmatic studies could evaluate outcomes related to pain and the use of antidepressants in this population.

## CONCLUSIONS

Different conditions negatively impact the health of women with cervical cancer, from sociodemographic aspects to clinical factors, whether in physical condition or psychological suffering. The RGI intervention is an economical and feasible approach that attests to the potential for symptomatic care in reducing anxiety in a complementary way. It is necessary to evaluate complications, recognize the limitations of conventional treatments, value and implement feasible interventions with a focus on integrative oncology.

Our study provided evidence that confirms the reduction of anxiety during concomitant radiochemotherapy treatment with the RGI intervention through RV. Although the safety of the intervention was not evaluated, no occurrences of adverse events or loss of follow-up were reported, and the technique is easily applicable in nursing practice.
